# First person – Jianhao Zeng

**DOI:** 10.1242/dmm.050510

**Published:** 2023-11-13

## Abstract

First Person is a series of interviews with the first authors of a selection of papers published in Disease Models & Mechanisms, helping researchers promote themselves alongside their papers. Jianhao Zeng is first author on ‘ A genetic mosaic mouse model illuminates the pre-malignant progression of basal-like breast cancer’, published in DMM. Jianhao conducted the research described in this article while a PhD student in Hui Zong's lab at University of Virginia Health System, Charlottesville, VA, USA. He is now a postdoc in the lab of Hamideh Parhiz at University of Pennsylvania, Philadelphia, PA, USA, investigating mRNA-based *in vivo* cellular reprogramming for cancer prevention and treatment.


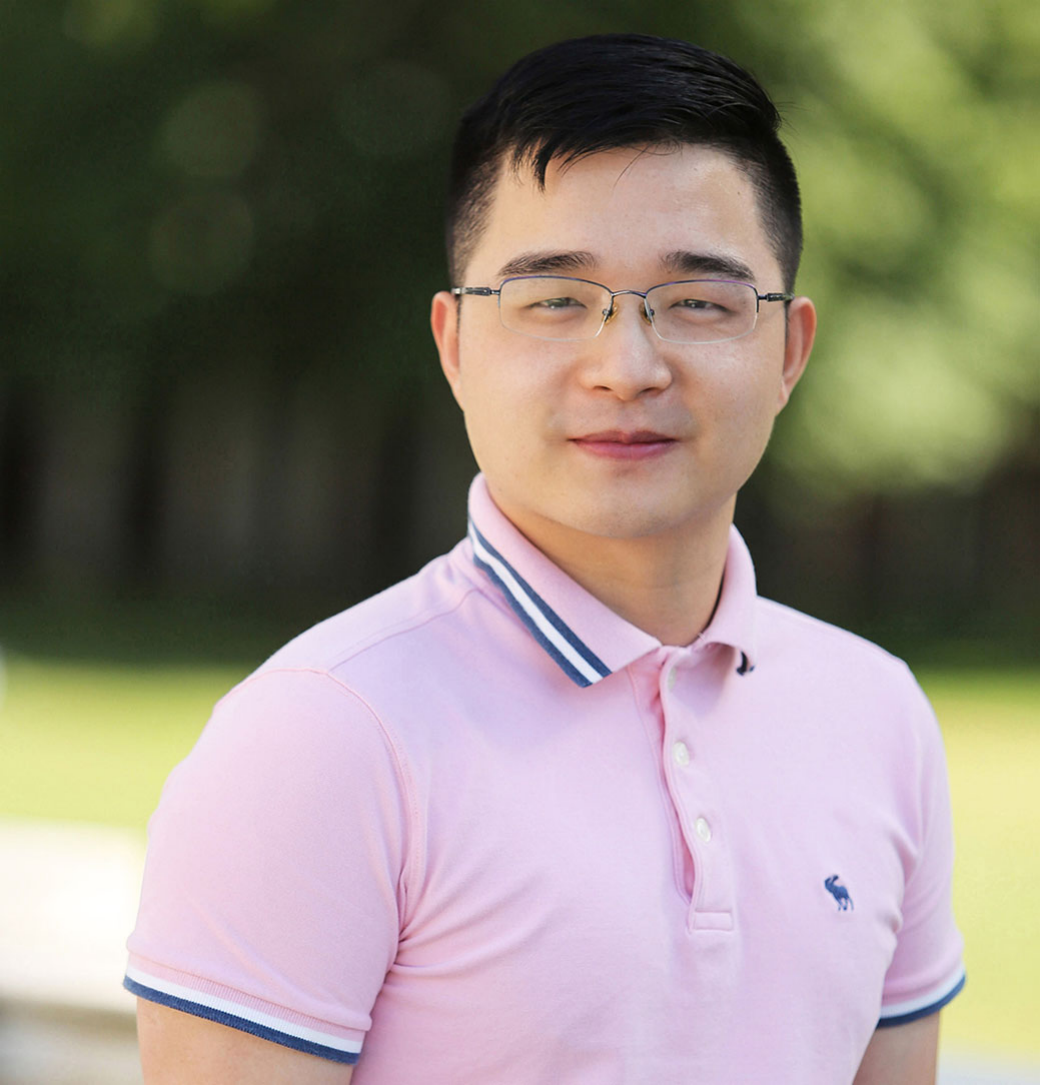


**Jianhao Zeng**

**How would you explain the main findings of your paper to non-scientific family and friends?**

Basal-like breast cancer is the most aggressive breast cancer subtype, with earlier onset, increased chance of metastasis and absence of hormonal-therapy targets. Detecting and intervening in cancer at an early stage can significantly improve patient outcomes, necessitating a comprehensive understanding of its pre-malignant development. However, studying cancer's pre-malignant stages in humans is challenging due to the limited number of cases detected at this early phase and, thus, restricted access to patient samples. To address this limitation, we've developed a mouse model that faithfully replicates the entire process of human basal-like breast cancer development, starting with sporadic mutant cells and culminating in malignant tumors. In this model, cancer-initiating cells are permanently labeled with a fluorescence reporter from the beginning, enabling the identification of mutant cells at various pre-malignant stages, irrespective of their normal pathological characteristics and morphology. Leveraging this new model, we have uncovered a stepwise pre-malignant progression trajectory of cancer, beginning with focal expansion, advancing to hyper-alveolarization, then to micro-invasions, and concluding with malignant tumors. Moreover, we've pinpointed a partial luminal-to-basal transitional state exclusively occurring in micro-invasive lesions. In essence, we have established a novel mouse model that offers exceptional spatiotemporal resolution for studying the pre-malignant progression of basal-like breast cancer. This invaluable resource provides a platform for the exploration and testing of early detection and cancer prevention strategies for basal-like breast cancer.

**What are the potential implications of these results for your field of research?**

Our study has introduced a mouse model that offers exceptional spatiotemporal resolution for investigating the pre-malignant stages of basal-like breast cancer, which should provide a valuable resource for pre-clinical research on early detection and cancer prevention. Importantly, while our study primarily focuses on breast cancer modeling, the MADM genetic module we utilized here can also be adapted for use in other cancer types. Leveraging this new mouse model, we have elucidated the progression trajectory of basal-like breast cancer, offering valuable insights into the early genesis of this disease. First, contrary to the initial belief that hyper-alveolarization of premalignant mammary cells stems from the abnormal expansion of mutant alveolar cells, our study demonstrates that it originates from mutant ductal cells, which likely undergo misdifferentiation towards an alveolar fate as the disease progresses. Second, at the micro-invasion stage, we observed that mutant cells undergo a partial luminal-to-basal transition – a crucial step known to be critical for complete transformation. The precise mapping of when this transitional event occurs provides a defined window for potential interventions.

**What are the main advantages and drawbacks of the experimental system you have used as it relates to the disease you are investigating?**

Compared to conventional genetically engineered mouse models for cancer, the MADM-based model we present here offers several unique advantages: (1) it generates a limited number of homozygous mutant cells from heterozygous mother cells, resembling cancer initiation in germline mutation carriers, where cancer begins with sporadic loss of heterozygosity; (2) the mutant cells are faithfully and permanently labeled with GFP immediately upon being generated, allowing mutant cells to be examined throughout the entire tumorigenesis; (3) the GFP labeling is sufficiently bright to visualize mutant cells in fresh, intact glands under a fluorescence stereomicroscope, allowing for gross pre-malignant status evaluation before further analyses requiring unfixed tissue or live cells; and (4) sibling wild-type cells labeled with RFP are generated simultaneously along the mutant cells, serving as an internal control for identifying even subtle abnormalities in mutant cells. Despite its strengths, the MADM-based model also has several limitations: (1) the establishment of the model involves complex breeding procedures, which is time consuming; (2) the progression from initiation to pre-malignant stages, as described in this study, spans approximately 8 months, restricting its feasibility for large-scale use; and (3) the current version of our mouse model has a mixed genetic background, which restricts its usage for allograft experiments. We are actively working on backcrossing this model into the FVB background to enhance the model's versatility.

[…] mammary epithelial cells with *p53* and *Brca1* loss maintained a luminal status until a very late stage of cancer development, before undergoing a partial luminal-to-basal transition.

**Figure:**
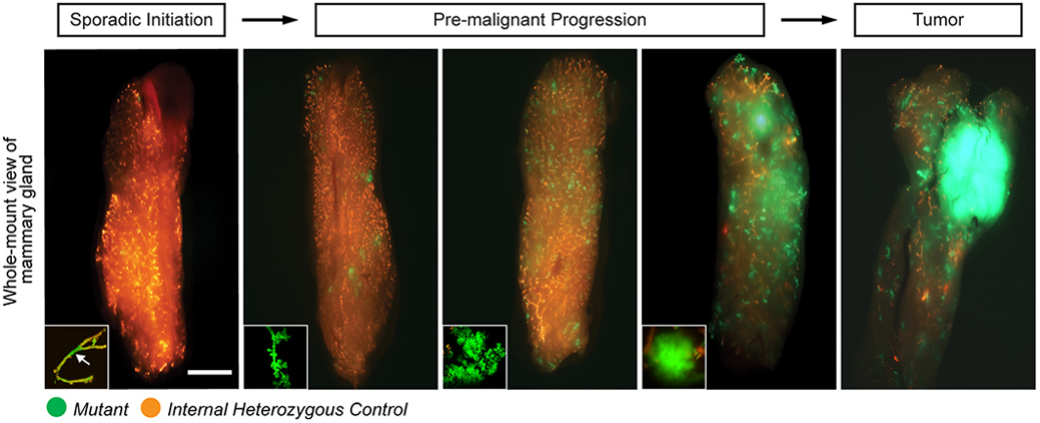
**Progressive morphological changes in mammary glands during cancer development.** Whole-mount fluorescence images of mammary glands from MADM-mutant mice of cohorts of age (left to right: ∼3, 6, 8, 10, 12 months; *n*=10), showing the progressive advancement of GFP^+^ mutant cells from initiation to tumor formation. Scale bar: 500 μm.

**What has surprised you the most while conducting your research?**

With our breast cancer mouse model, I was surprised to observe that mammary epithelial cells with *p53* and *Brca1* loss maintained a luminal status until a very late stage of cancer development, before undergoing a partial luminal-to-basal transition. This observation contrasts with previous *in vitro* studies, which suggested that *p53* and/or *Brca1* loss alone was sufficient to induce the luminal-to-basal transition of mammary epithelial cells. Our data imply that additional triggers may be needed for this critical cell state transition, which is known to play a critical role in the progression of breast cancer partly by increasing cellular stemness and invasiveness. Further efforts to identify those additional triggers could unveil new targets for suppressing cancer progression.

**What do you think is the most significant challenge impacting your research at this time and how will this be addressed over the next 10 years?**

While intervening in cancer at its premalignant stages holds immense promise, the most critical obstacle lies in accurately detecting early-stage disease. It is imperative to attain a level of accuracy that can unequivocally justify interventional treatments for asymptomatic individuals, a need currently unmet for many types of cancer. Advancements in high-throughput sequencing and mass spectrometry have greatly enhanced our ability to detect minute quantities of circulating tumor DNA/proteins in the bloodstream. When coupled with the power of artificial intelligence for data analysis to identify strong correlations between blood components and early-stage cancer, we can anticipate the emergence of highly precise approaches for cancer early detection in the coming decade.

As a postdoc, the conventional career path often presents a binary choice: pursue a tenure-track professorship or exit academia.

**What changes do you think could improve the professional lives of scientists?**

As a postdoc, the conventional career path often presents a binary choice: pursue a tenure-track professorship or exit academia. While I am deeply passionate about research and aspire to continue my academic journey, I am less enthusiastic about the extensive responsibilities that come with managing a research group and the constant demand for grant writing. I firmly believe that academia should offer more intermediate-level positions tailored to individuals like me, such as senior research scientist roles. These positions allow scientists to focus primarily on their research endeavors, providing greater career stability compared to postdoc positions, while also being less demanding on the aspects of management compared to a professorship. The scarcity of such positions is, in part, due to the inadequate availability of long-term funding to support this role. Establishing more of these positions could help retain highly skilled scientists within academia and offer a valuable alternative to the binary choice currently in place for postdocs.

**What's next for you?**

I've recently started a postdoctoral position at the University of Pennsylvania, where my lab specializes in using cutting-edge mRNA delivery techniques for cell reprogramming. These techniques have gained prominence through their success in COVID vaccines. Leveraging these techniques, my focus is on targeting crucial transitional events in cancer pre-malignancy, such as the partial luminal-to-basal transitional state of breast premalignant cells we've identified in this study. Our goal is to reprogram these cells and potentially intercept cancer at its early stages. I'm thrilled about my new research journey ahead.
